# Sorafenib inhibits intracellular signaling pathways and induces cell cycle arrest and cell death in thyroid carcinoma cells irrespective of histological origin or *BRAF* mutational status

**DOI:** 10.1186/s12885-015-1186-0

**Published:** 2015-03-26

**Authors:** Martina Broecker-Preuss, Stefan Müller, Martin Britten, Karl Worm, Kurt Werner Schmid, Klaus Mann, Dagmar Fuhrer

**Affiliations:** 1 Department of Endocrinology and Metabolism, and Division of Laboratory Research, University Hospital Essen, Hufelandstr. 55, Essen, Germany; 2 Department of Nuclear Medicine, University Hospital Essen, Hufelandstr. 55, Essen, Germany; 3 Institute of Pathology at the University Hospital Essen, Hufelandstr. 55, Essen, Germany; 4 Present address: Department of Clinical Chemistry, University Hospital Essen, Hufelandstr. 55, 45122 Essen, Germany; 5 Present address: University Hospital Essen, Klinik für Anästhesiologie & Intensivmedizin, Hufelandstr. 55, 45122 Essen, Germany; 6 Present address: Center of Endocrinology Alter Hof München, Dienerstr. 12, 80331 München, Germany

**Keywords:** Dedifferentiated thyroid carcinoma, Sorafenib, Multi-kinase inhibitor, Molecular targeted therapy, BRAF mutation, MAP kinase

## Abstract

**Background:**

Patients with dedifferentiated or anaplastic thyroid carcinomas currently lack appropriate treatment options. Kinase inhibitors are among the most promising new agents as alternative strategies. The BRAF- and multi-kinase inhibitor, sorafenib, has already shown antitumor effects in thyroid carcinoma patients in a phase III clinical trial. In this study we aim to better characterize molecular effects and efficacy of sorafenib against thyroid carcinoma cells with various histological origins and different *BRAF* mutational status. Analysis of different signaling pathways affected by sorafenib may contribute to assist a more specific therapy choice with fewer side effects. Twelve thyroid carcinoma cell lines derived from anaplastic, follicular and papillary thyroid carcinomas with wildtype or mutationally activated BRAF were treated with sorafenib. Growth inhibition, cell cycle arrest, cell death induction and inhibition of intracellular signaling pathways were then comprehensively analyzed.

**Methods:**

Cell viability was analyzed by MTT assay, and the cell cycle was assessed by flow cytometry after propidium iodide staining. Cell death was assessed by lactate dehydrogenase liberation assays, caspase activity assays and subG1 peak determinations. Inhibition of intracellular pathways was analyzed in dot blot and western blot analyses.

**Results:**

Sorafenib inhibited proliferation of all thyroid carcinoma cell lines tested with IC50 values ranging between 1.85 and 4.2 μM. Cells derived from papillary carcinoma harboring the mutant *BRAF*^*V600E*^ allele were slightly more sensitive to sorafenib than those harboring wildtype *BRAF*. Cell cycle analyses and caspase assays showed a sorafenib-dependent induction of apoptosis in all cell lines, whereas increased lactate dehydrogenase release suggested cell membrane disruption. Sorafenib treatment caused a rapid inhibition of various MAP kinases in addition to inhibiting AKT and receptor tyrosine kinases.

**Conclusions:**

Sorafenib inhibited multiple intracellular signaling pathways in thyroid carcinoma cells, which resulted in cell cycle arrest and the initiation of apoptosis. Sorafenib was effective against all thyroid carcinoma cell lines regardless of their tumor subtype origin or *BRAF* status, confirming that sorafenib is therapeutically beneficial for patients with any subtype of dedifferentiated thyroid cancer. Inhibition of single intracellular targets of sorafenib in thyroid carcinoma cells may allow the development of more specific therapeutic intervention with less side effects.

## Background

Thyroid carcinoma originating from thyroid follicular cell is the most common endocrine malignancy [1,2]. About 90% of thyroid carcinomas are well differentiated, while 10% or less are poorly differentiated or anaplastic subtypes [2,3]. Of the differentiated carcinomas, 85 to 90% are papillary and 10 to 15% follicular subtypes. Most differentiated carcinomas progress slowly, and patients usually become disease-free after initial treatment with thyroidectomy and radioiodine ablation. In contrast, 10 to 15% of patients initially diagnosed with differentiated carcinomas experience recurrent disease [1,4,5]. A reduction in radioiodine uptake and storage accompanies tumor dedifferentiation. Dedifferentiated tumors are more aggressive and lead to a worse patient outcome [3,5,6]. Tumors initially categorized as poorly differentiated (PDTC) or anaplastic thyroid carcinomas (ATC) share these features early on. Anaplastic (undifferentiated) thyroid carcinomas are highly aggressive and lethal tumors that have completely lost the ability to take up iodine [7]. Beside their aggressive growth particularly the loss of capacity to uptake iodine makes both dedifferentiated and anaplastic thyroid carcinomas difficult to treat, and confer the poor patient prognosis. Moreover, chemotherapeutic treatment proved to be largely ineffective against aggressive thyroid carcinomas [8]. These inadequacies of current treatment protocols for dedifferentiated and anaplastic thyroid carcinomas strongly emphasize the urgent need to establish novel targeted treatment options.

A better understanding of the molecular alterations driving thyroid tumorigenesis can drive development of appropriate targeting agents for thyroid carcinoma. Mutations in genes encoding the proteins of the mitogen activated protein (MAP) kinase signaling cascade (RAS-RAF-mitogen-activated protein kinase kinase (MEK)- extracellular-signal regulated kinase (ERK)) frequently occur in thyroid carcinomas [2,3]. About 50% of papillary thyroid carcinomas (PTC) harbor activating mutations in the *BRAF* gene (mostly *BRAF*^*V600E*^), an effector of MEK that in turn activates the ERK1 and ERK2 mitogen-activated protein kinases (Review [9,10]). *BRAF* mutations also occur in up to 13% of PDTCs and 35% of ATCs [11], but in these subtypes are restricted to tumors with a papillary component or supposed to be derived from PTC [12]. The *BRAF*^*V600E*^ mutation has been associated with advanced clinical stage, loss of iodine accumulation and has an independent prognostic value for PTC recurrence [13,14]. Mutations in the three *RAS* genes, *HRAS, KRAS* and *NRAS*, have been described in all thyroid epithelial carcinoma subtypes (Review [3]). Besides direct mutational activation of the RAS-RAF-MEK-ERK signaling pathways, receptors with intrinsic tyrosine kinase activity can also stimulate this cascade. Overexpression and autocrine activation of the epidermal growth factor receptor (EGFR) in thyroid carcinomas contributes to the activation of the RAS-MAP kinase cascade [15,16]. Expression of the platelet-derived growth factor receptors (PDGFR) and their ligands in undifferentiated thyroid cells [17,18] also activates this cascade. An aberrant activation of the RAS-RAF-MEK-ERK signaling cascade, therefore, is common in all thyroid carcinoma subtypes, and may provide targets for appropriate molecular therapies.

Inappropriate activation of the MEK-ERK kinase cascade leads to deregulated cell proliferation, dedifferentiation and improved cell survival in a variety of tumor cell types [19]. The importance of this pathway and its frequent deregulation and mutational activation in cancers has led to development of small molecule inhibitors. One of these inhibitors is sorafenib (Nexavar®, BAY43-9006), which was originally designed to inhibit the ARAF, BRAF and RAF1 kinases [20]. Sorafenib competitively inhibits ATP binding to RAF catalytic domains, thus, inhibiting kinase activity via stabilization of the conserved kinase domain in the inactive configuration [21]. Sorafenib was shown to potently inhibit RAF1 kinase, wildtype BRAF and oncogenic BRAF^V600E^ in vitro [22]. Moreover, sorafenib directly blocks the autophosphorylation and activation of several receptor tyrosine kinases, including PDGFRB, fibroblast growth factor receptor 1 and vascular endothelial growth factor receptors (VEGFRs) [20]. Sorafenib decreases ERK activation in human tumor cells, inhibits cell proliferation in vitro and inhibits growth of human tumor xenografts in nude mice [20,23,24]. Sorafenib has been shown to inhibit RAF activation, phosphorylation of members of the MEK-ERK kinase family and proliferation of cell lines derived from PTC and ATC harboring an activating *BRAF* mutation [25]. These effects were similar after BRAF knockdown using siRNA, suggesting a central role for mutationally activated BRAF [25]. Furthermore, Carlomago et al. [26] showed that sorafenib inhibits RET kinase and thus proliferation of papillary and medullary thyroid carcinoma cells harboring an oncogenic RET kinase. Sorafenib treatment inhibited proliferation and improved survival of mice with ATC xenografts [27]. Taken together, these results demonstrate the efficacy of sorafenib against various cell lines derived from PTCs and ATCs. However, current published reports include no data directly comparing cell lines with and without *BRAF* mutations or describing the effects of sorafenib in cell lines derived from follicular thyroid carcinomas (FTC).

Some clinical phase II trials and clinical studies in patients with metastatic differentiated thyroid carcinomas have shown promising results for sorafenib [28-32]. The majority of these studies detected no differences in treatment efficacy between thyroid carcinoma subtypes, although the low case numbers in these studies may have hindered subgroup analysis. Positive effects were reported in one phase II trial in patients with advanced ATC, which showed partial responses in 2 of 20 patients and stable disease in 5 of 20 patients [33]. A recently published phase III multicenter, double-blind randomized and placebo-controlled trial evaluating the efficacy of sorafenib in thyroid cancer patients (DECISION study) [34,35] demonstrated that sorafenib significantly improved progression-free survival compared with placebo in patients with progressive radioiodine-refractory differentiated thyroid cancer independent of the clinical and genetic subgroup. Overall, sorafenib has exhibited significant antitumor activity and clinical benefits in patients with progressive and advanced thyroid carcinoma and thus is a treatment option for patients with locally recurrent or metastatic, progressive, differentiated thyroid carcinoma refractory to radioactive iodine treatment.

Since sorafenib as a multikinase inhibitor blocks various intracellular signaling pathways, significant side effects have also been reported in clinical trials [36]. A broader analysis of the signaling molecules affected by sorafenib treatment in specific tumor cell types may thus be useful to identify cell-specific key signaling molecules for more directly targeted treatment approaches. No data are currently available on the intracellular effects of sorafenib in thyroid carcinoma cells or potential differences in sorafenib action in thyroid carcinoma cells of the papillary (with or without the *BRAF*^*V600E*^ mutation), follicular or anaplastic subtypes. The aim of the present study was to elucidate the effects of sorafenib treatment on proliferation, cell death induction and intracellular signaling pathways in various thyroid carcinoma cell lines.

## Methods

### Compounds and antibodies

Sorafenib (BAY 43–9006, Nexavar®) was provided by Bayer Health Care (Wuppertal, Germany), stored in 10 mM aliquots in DMSO at −20°C and further diluted in the appropriate medium. Antibodies to detect both total protein and activated phosphorylated forms of c-Jun N-terminal kinase (JNK), AKT, p44/42 MAP kinase (ERK1/2) and p38 MAPK were purchased from Cell Signaling Technology (Danvers, MA, USA).

### Cell lines and cell culture

Cell lines derived from the anaplastic, papillary and follicular thyroid cancer subtypes were used in this study. The SW1736 [37], HTh7 [38], HTh74 [39], HTh83 [40], and C643 [17] cell lines were derived from ATC. BHT101 [41], B-CPAP [42], and TPC [43] cell lines were derived from PTC. ML1 [44] and TT2609 [45] are FTC-derived cell lines. The FTC133, FTC236 and FTC238 [46] cell lines were derived from a single primary FTC, a lymph node metastasis and a lung metastasis from the same patient, respectively. The HTh7, HTh74, HTh83, C643 and SW1736 cell lines were a gift from Prof. Heldin (Uppsala, Sweden), and all other cell lines were purchased from ATCC (Manassas, VA, USA), ECACC (Salisbury, UK) and DSMZ (Braunschweig, Germany). Cell lines were maintained in their appropriate media supplemented with 10% fetal bovine serum (FBS, Life Technologies, Paisley, PA, USA) at 37°C at 5% CO_2_.

### DNA extraction and mutation analysis

Genomic DNA was isolated from cell lines using the QIAamp DNA kit (Qiagen, Hilden, Germany) according to the manufacturer’s instructions. Primers used to amplify exon 15 of the *BRAF* gene were described elsewhere [47]. For PCR amplification, 5 μl of DNA solution containing 200 ng DNA was used in a 50 μl reaction containing 1xPCR buffer, 1.5 mM MgCl_2_, 1.5U HotMaster Taq polymerase (Eppendorf, Hamburg, Germany) and 300 nM each of forward and reverse primers. Cycling conditions were 40 cycles of 94°C for 20 sec, 55°C for 10 sec, 65°C for 35 sec. PCR products were analyzed on 3% agarose gels and purified using the QIA quick removal kit (Qiagen). Sequencing was performed using the ABI Prism BigDye Terminator Cycle sequencing kit v1.1 on an ABI Prism 3100 Genetic Analyzer (Applied Biosystems, Foster City, CA, USA). Sequences were compared to the wildtype sequences using the Sequencher software (Gene Codes, Ann Arbor, MI, USA).

### Cell proliferation studies

For proliferation assays, 1 × 10^4^ to 5 × 10^4^ cells (cell line dependent) were seeded into 96-well plates containing the appropriate growth medium. Medium was replaced after 24 hours with culture medium without FBS but containing 0.1% bovine serum albumin (BSA) and the indicated sorafenib concentrations was added. After 48 hours, viable cells were stained with the Cell Titer Aqueous One Solution assay (Promega, Madison, WI, USA), and optical density at 490 nm was measured using an Emax microplate photometer (Molecular Devices, Sunnyvale, CA, USA). Control values without sorafenib treatment were performed as 22-fold determinations, while all concentrations of sorafenib were tested in 8-fold. Calculation of results and Student’s t-test were performed using SoftMax pro software (Molecular Devices), and IC50 values were calculated using Sigma Plot software (Systat, San Jose, CA, USA).

### Determination of lactate dehydrogenase release and caspase 3/7 activity measurement

Release of lactate dehydrogenase (LDH) from cells with damaged membranes was measured by the CytoTox-ONE homogeneous membrane integrity assay (Promega). Activity of caspases 3 and 7 was measured by the Apo-ONE homogeneous Caspase 3/7 assay (Promega). 1 × 10^4^ to 5 × 10^4^ cells (cell line dependent) were seeded into black, transparent-bottomed 96-well plates containing the appropriate growth medium. Medium was removed after 24 h and 100 μl culture medium without FBS, but containing 0.1% BSA and the denoted sorafenib concentration, was added to each well. After 14 or 24 hours, 50 μl of medium from each well was transferred to a fresh black 96-well plate and equilibrated to 20°C. According to the manufacturer’s instructions, 50 μl of CytoTox reagent was added and reactions were incubated for 10 min in the dark. After adding 25 μl of stop solution, fluorescence was determined with excitation and emission wavelengths of 560 nm and 590 nm, respectively. Wells containing no cells, as the zero setting, and fully lysed cells, as the maximum LDH release control, were included in each experiment. Caspase 3 and 7 activity in treated cells was determined in the original stimulation plate by adding 50 μl of Apo-ONE reagent that contained a fluorometric substrate in cell lysis and reagent buffer. After 60 min, fluorescence was measured at 521 nm after excitation with 499 nm. All values were performed as 8-fold determinations. Calculation of results and Student’s t-tests were performed using SoftMax pro software (Molecular Devices).

### Cell cycle analysis

Cells were plated at 1 × 10^5^ to 5 × 10^5^ cells/well in 6-well plates in appropriate growth medium for cell cycle analyses. Medium was replaced with medium without FBS but containing 0.1% BSA and 3 μM sorafenib 24 h later and cells were treated for the indicated times. Treated cells were harvested and fixed in cold 70% ethanol. RNase A (60 μg/ml) and propidium iodide (25 μg/ml) in PBS were added, and samples were incubated 20 minutes in the dark at room temperature. Samples were measured on a FACS Calibur flow cytometer (Becton Dickinson, San Jose, CA), and cell cycle stages were analyzed using the ModFit Software (Verity Software House, Topsham, ME, USA).

### Proteome Profiler™ array and western blot analysis

Proteome Profiler™ antibody arrays (R&D systems, Mineapolis, MN, USA) and western blotting were used to assess inhibitory effects of sorafenib on intracellular signaling proteins and receptor tyrosine kinases. Cells were plated in 10 cm culture dishes, and grown for 1–2 days to 85 to 90% confluency. Medium was removed and cells were washed once and maintained in prewarmed HBSS buffer (Life Technologies) for 20 minutes before adding 3 μM sorafenib. Treated cells were washed with ice-cold PBS, and all further steps were performed on ice. Cells were lysed in lysis buffer containing cOmplete protease inhibitor and phosSTOP phosphatase inhibitor cocktails (Roche Applied Science, Mannheim, Germany). Lysates were clarified by centrifugation at 10,000 × g for 10 min at 4°C, protein concentration determined by modified Bradford assay (Bio-Rad Laboratories, Hercules, CA, USA) and 500 μg of protein from each lysate were used in dot blot analysis according to the manufacturer’s instructions. For western blotting, 30 μg of total protein was denatured by boiling for 5 minutes in SDS sample buffer, then separated by SDS-PAGE and transferred to nitrocellulose membranes (Bio-Rad Laboratories). After blocking with 5% skim milk powder or 5% BSA in TBS, blots were incubated with the appropriate primary antibody in TBS buffer containing 0.1% Triton X-100 (TBS-T) overnight at 4°C. After washing, an appropriate secondary antibody coupled to horseradish peroxidase in TBS-T was added. Bound antigens on western and dot blots were detected using the ECL Advance chemiluminescence detection kit (GE Healthcare, Piscataway, NJ, USA). Signal intensity was evaluated with a CCD camera system, and differences were calculated with the Quantity One software (Bio-Rad Laboratories).

### Statistical analysis

Statistical analysis of treatment versus control groups was performed by means of the unpaired Student’s t-test using SPSS (IBM Inc, Armonk, NY, USA) or the other software packages indicated above. P-values < 0.05 were considered statistically significant.

## Results

### Sorafenib inhibited proliferation of cell lines derived from all thyroid tumor subtypes irrespective of BRAF status

To assess whether sorafenib has a selective effect on proliferation of cells with different histological and molecular thyroid carcinoma backgrounds, we treated 12 cell lines with different histological origins and *BRAF*^*V600E*^ mutational status for 48 h with a range of sorafenib concentrations or vehicle and assessed proliferative activity. We first assessed the mutational status of exon 15 of the *BRAF* gene in all cell lines using PCR. The BHT101 and B-CPAP papillary cell lines both harbored a heterozygous *BRAF*^*V600E*^ mutation. The anaplastic cell line, SW1736 also harbored a heterozygous *BRAF*^*V600E*^ mutation which is suggestive that the anaplastic tumor has originated from a papillary carcinoma (Table 1). Only wildtype *BRAF* alleles were detected in the TPC1 papillary cell line, the C643, HTh7 and HTh83 anaplastic cell lines and the FTC133, FTC236, FTC238, ML1 and TT2609 follicular cell lines (Table 1).

**Table 1:** **Cell line characteristics,** ***BRAF*** ^***V600E***^ **mutational status and viability after sorafenib treatment for 48 hours of all thyroid carcinoma cell lines examined**

Cell line	Origin	*BRAF*^*V600E*^-mutation	IC50 sorafenib (μM)	Lowest effective concentration (μM)
BHT101	Papillary	Heterozygous	2.1	1.0
B-CPAP	Papillary	Heterozygous	1.85	1.0
TPC1	Papillary	No	2.6	0.05
FTC133	Follicular	No	2.9	1.0
FTC236	Follicular	No	3.2	0.5
FTC238	Follicular	No	4.2	0.01
ML1	Follicular	No	2.95	2.0
TT2609	Follicular	No	3.05	1.0
SW1736	Anaplastic	Heterozygous	3.25	1.0
HTh83	Anaplastic	No	3.95	2.0
C643	Anaplastic	No	3.2	0.01
HTh7	Anaplastic	No	3.1	2.0

**Figure 1:**
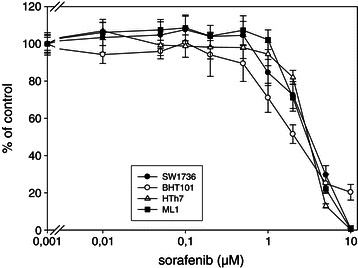
**Sorafenib reduced the viability of thyroid carcinoma cell lines of different histological derivation.** Cells were cultured with increasing concentrations of sorafenib or vehicle (DMSO) control for 48 h, and viability was assessed by MTT assay. Values are reported as percent of vehicle control ± standard deviation, and represent mean values of eight determinations of one representative experiment of three. IC50 values and the lowest concentration that caused a significant loss of viability for all cell lines examined are depicted in Table 1.

Sorafenib treatment decreased the number of viable cells in all 12 thyroid carcinoma cell lines analyzed, and efficiency of sorafenib did not span a large range, since IC50 values for all 12 cell lines were between 1.85 μM and 4.2 μM (Table 1). The BHT101 and B-CPAP papillary cell lines, which harbor the *BRAF*^*V600E*^ mutation had the lowest IC50 values (2.1 and 1.85 μM), while SW1736 cells (anaplastic cell line with *BRAF*^*V600E*^ mutation) had a midrange IC50 of 3.25 μM. TPC1 cells, which are derived from PTC but harbor no *BRAF*^*V600E*^ mutation, had an IC50 value in the lower range that was slightly higher than those of the other 2 papillary cell lines, which harbor *BRAF*^*V600E*^ mutations. The follicular cell lines, FTC133, FTC236, ML1 and TT2609, and the C643 and HTh7 ATC cell lines also had midrange IC50 values (2.9-3.2 μM and 3.1-3.2 μM, respectively). HTh83 ATC cells and FTC238 FTC cells were most insensitive to sorafenib, with IC50s of 3.95 and 4.2 μM, respectively. While no dramatic differences were observed in the sensitivity of cell lines from different histological origins or with or without the *BRAF*^*V600E*^ activating mutations to sorafenib, some trends were observed. The three papillary cell lines had the lowest overall IC50 values, and the two papillary cell lines harboring the *BRAF*^*V600E*^ mutation (BHT101 and B-CPAP) were slightly more sensitive than TPC1 cells. However, the only *BRAF*^*V600E*^ mutation harboring anaplastic cell line (SW1736) had an IC50 in midrange of the IC50s for all 4 anaplastic cell lines. These results indicate that BRAF activation does not play any role in undifferentiated carcinoma cells. Cell lines from follicular carcinomas, with exception of the relatively insensitive FTC238 cells, had midrange IC50 values suggesting that sorafenib targets kinases other than BRAF in these cells. Results for one representative cell line of all histological origins with or without *BRAF*^*V600E*^ mutation (SW1736 cells (anaplastic with *BRAF*^*V600E*^ mutation), HTh7 (anaplastic without *BRAF*^*V600E*^ mutation), BHT101 (papillary with *BRAF*^*V600E*^ mutation) and ML1 (follicular without *BRAF*^*V600E*^ mutation) are depicted in Figure 1.

In addition to determination of IC50 values, for each experiment we noted the lowest sorafenib concentration that significantly inhibited cell viability compared to unstimulated controls (Table 1). Interestingly, the lowest effective sorafenib concentration was in a wide range in all cell lines examined (0.05 to 2.0 μM; Table 1). It was the lowest in FTC238 (follicular cell line), C643 (anaplastic) and TPC1 (papillary cell line without *BRAF*^*V600E*^ mutation) cells (0.01 μM and 0.05 μM sorafenib; Table 1). HTh7 and HTh83 (both anaplastic cell line without *BRAF*^*V600E*^ mutation) and ML1 follicular cells were the most insensitive cell lines with respect to the lowest effective concentration of sorafenib (2.0 μM; Table 1). Taken together, sorafenib treatment effectively inhibited viability of all twelve cell lines with different histological and molecular thyroid tumor backgrounds, producing IC50 values ranging from 1.85 to 4.2 μM. The presence of the activating *BRAF*^*V600E*^ mutation appeared to render cell lines derived from the more differentiated papillary tumors slightly more suseptible to sorafenib, while activated BRAF in SW1736 cells derived from anaplastic tumors had no effect on sorafenib efficacy.

### Sorafenib increased the proportion of cells in subG1 peak and induced cell cycle arrest in thyroid carcinoma cells

**Figure 2:**
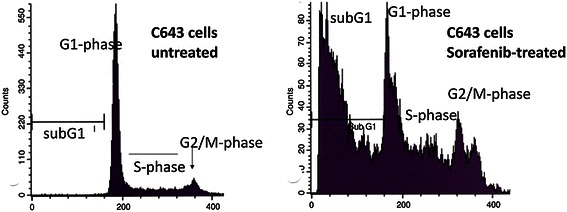
**Cell cycle changes in C643 cells before and after incubation with 3 μM sorafenib for 24 h hours.** Cell cycle analysis was conducted using FACS, and this figure shows the complete results for one cell line as an example. Besides the increase in SubG1 peak, in the remaining living cells a decrease in G1 phase and in G2/M-phase and an increase in S-phase of cell cycle was observed. Values for the other cell lines examined are depicted in Table 2.

To investigate the effects of sorafenib on cell cycle distribution and on cell death-associated DNA fragmentation, the 12 cell lines were analyzed flow cytometrically after propidium iodide staining following sorafenib treatment. The subG1 fraction increased markedly in all cell lines analyzed after 24 h treatment with 3 μM sorafenib, indicating that sorafenib induced cell death and DNA fragmentation (Figure 2 and Table 2). The percentage of cells in the subG1 peak was the highest in TPC1 papillary (72.4%) and HTh83 anaplastic cells (74.4%). Increases were lowest in the subG1 peaks of TT2609 (21.5%) and FTC133 (22.1%) follicular cells and HTh7 anaplastic cells (25.7%), but were still significant. Cell lines derived from PTC appeared most susceptible to cell death induction by sorafenib, with the highest percentages of cells in subG1 after treatment (60.2 to 72.4%). Sorafenib had the most variable effect on anaplastic cell lines, increasing the subG1 fraction in HTh7 cells by 25.7% and in HTh83 cells by 74.7%. In follicular cell lines percentage of subG1 fraction varies from 21.5% in TT2609 to 43.0% in FTC238 cells. Presence of the activating *BRAF*^*V600E*^ mutation appeared not to influence the ability of sorafenib to induce DNA fragmentation in cells of various histological origins. In the cells that did not enter subG1, sorafenib appeared to have varying effects on the cell cycle. Sorafenib treatment increased the proportion of cells in G1 and decreased the proportion of cells in S phase in all papillary cell lines (BHT101, B-CPAP and TPC1) and in the SW1736 and HTh7 anaplastic cell lines (Figure 2 and Table 2). Sorafenib treatment had the opposite effect on the C643 anaplastic cell line and the FTC133, FTC236 and FTC238 follicular cell lines, which responded by increasing numbers in S phase and decreasing numbers in the G1 phase. Sorafenib caused an increase in the proportion of ML1 follicular cells and HTh83 anaplastic cells in the G2/M phase accompanied by fewer cells in S phase, while the cell cycle distribution in TT2609 follicular cells was not significantly altered.

**Table 2:** **Percentage of thyroid carcinoma cells determined by FACS analysis in each cell cycle phase following 24 h of treatment with sorafenib or vehicle**

Cell line	Type	Status	%SubG1	%G1	%G2/M	%S
BHT101	Papillary	Unstimulated	4.5 ± 0.6	58.3 ± 3.9	17.7 ± 0.9	24.0 ± 1.3
		Sorafenib 24 h	60.2 ± 6.4*	72.1 ± 5.8*	13.4 ± 0.7*	14.5 ± 0.9*
B-CPAP	Papillary	Unstimulated	6.8 ± 2.3	57.1 ± 2.7	14.0 ± 1.1	28.9 ± 1.4
		Sorafenib 24 h	62.3 ± 7.3*	69.8 ± 4.6*	23.1 ± 1.2*	7.1 ± 0.4*
TPC1	Papillary	Unstimulated	3.1 ± 0.4	48.1 ± 3.5	25.3 ± 1.6	26.6 ± 1.3
		Sorafenib 24 h	72.4 ± 5.9*	57.6 ± 4.2*	27.6 ± 3.5	14.8 ± 0.8*
FTC133	Follicular	Unstimulated	1.5 ± 0.3	57.0 ± 3.5	13.7 ± 0.8	29.3 ± 1.5
		Sorafenib 24 h	22.1 ± 3.4*	46.9 ± 4.4*	8.7 ± 0.5*	44.4 ± 2.9*
FTC236	Follicular	Unstimulated	1.2 ± 0.2	64.1 ± 5.0	12.1 ± 0.9	23.8 ± 1.1
		Sorafenib 24 h	35.2 ± 3.7*	36.0 ± 3.1*	15.6 ± 1.0*	48.4 ± 3.7*
FTC238	Follicular	Unstimulated	0.7 ± 0.1	46.1 ± 2.9	9.3 ± 0.6	44.6 ± 5.8
		Sorafenib 24 h	43.0 ± 5.0*	24.2 ± 1.4*	11.0 ± 1.7	64.6 ± 6.6*
ML1	Follicular	Unstimulated	1.0 ± 0.1	56.5 ± 3.2	18.1 ± 1.2	25.4 ± 1.3
		Sorafenib 24 h	40.4 ± 3.2*	59.9 ± 2.7	25.5 ± 1.8*	14.4 ± 0.8*
TT2609	Follicular	Unstimulated	1.6 ± 0.2	57.6 ± 4.6	11.5 ± 1.3	30.9 ± 1.8
		Sorafenib 24 h	21.5 ± 3.0*	54.7 ± 6.0	13.1 ± 1.6	32.2 ± 3.1
SW1736	Anaplastic	Unstimulated	2.2 ± 0.3	46.0 ± 2.9	10.1 ± 1.1	43.9 ± 2.7
		Sorafenib 24 h	53.6 ± 3.9*	68.1 ± 5.5*	9.7 ± 0.7	22.2 ± 1.2*
C643	Anaplastic	Unstimulated	1.0 ± 0.1	50.5 ± 3.7	15.1 ± 0.8	34.4 ± 2.8
		Sorafenib 24 h	43.8 ± 4.0*	38.6 ± 2.6*	11.5 ± 0.5*	49.9 ± 3.5*
HTh7	Anaplastic	Unstimulated	5.9 ± 0.8	52.2 ± 3.5	14.5 ± 0.8	33.3 ± 1.8
		Sorafenib 24 h	25.7 ± 3.4*	76.9 ± 6.4*	1.2 ± 0.1*	21.9 ± 0.9*
HTh83	Anaplastic	Unstimulated	5.6 ± 0.3	36.5 ± 2.6	24.3 ± 1.7	39.2 ± 2.0
		Sorafenib 24 h	74.7 ± 5.7*	35.0 ± 5.2	34.5 ± 1.9*	30.5 ± 1.5*

Values for subG1 peaks represent the percentage of all cells measured, while values for G1-, G2/M- and S-phase are depicted for the remaining living cells. Values are given as mean values ± standard deviation of 6-fold determinations. *indicates significant changes (p<0.05, Student’s t-test).

### Sorafenib induced cell death in thyroid carcinoma cells

**Figure 3:**
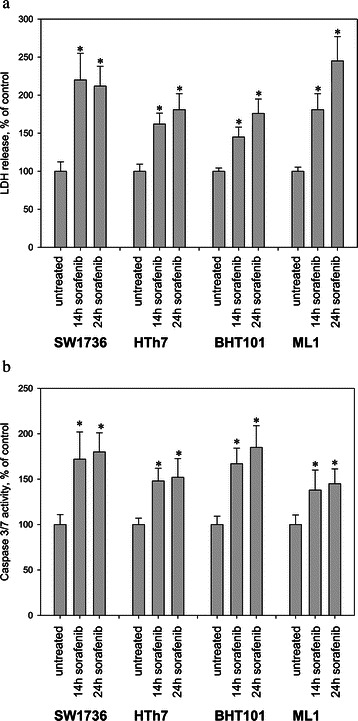
**Sorafenib induces cell death in thyroid carcinoma cell lines.** SW1736, HTh7, BHT101 and ML1 cells were incubated for 14 h and 24 h with 3 μM sorafenib or vehicle (DMSO). LDH release into the cell culture medium was measured using the Cytotox assay **(a)**, and increased caspase 3 and 7 activity was detected using the ApoOne assay **(b)**. Data represent mean values of eight-fold determinations ± standard deviation, and are depicted as percent of vehicle-treated control. *indicates significant increase (p<0.05, Student’s t-test).

To follow up on our detection of the decrease of viable cells and the increase of cells in subG1 after sorafenib treatment, we analyzed cell death in one cell line each derived from the papillary and follicular and in two cell lines derived from the anaplastic thyroid tumor subtypes. We monitored release of LDH into the culture medium, which results from the disruption of cell membranes and release of LDH with other cytoplasmic components. BHT101 papillary cells, ML1 follicular cells and SW1736 and HTh7 anaplastic cells were treated for either 14 h or 24 h with sorafenib before measuring LDH in the culture medium. LDH was significantly elevated in the culture medium from all four cell lines after sorafenib treatment compared to controls treated with only DMSO carrier concentrations (Figure 3a). The LDH levels released by SW1736 and ML1 cells after 24 h of treatment were slightly higher than levels released by HTh7 and BHT101 cells. Elevated LDH activities therefore reflected cell membrane disruption after sorafenib treatment. To assess whether cell death was due to apoptotic mechanisms, we assessed activity of the caspases 3 and 7 after sorafenib treatment. Caspase activities were significantly elevated after both 14 h and 24 h of sorafenib treatment in all four thyroid carcinoma cell lines (Figure 3b). Elevations in caspase 3 and 7 activities were nearly the same after either 14 h or 24 h of treatment in all four cell lines, suggesting an early activation of the apoptotic machinery by sorafenib. Overall, sorafenib not only decreased the number of viable cells and inhibited the cell cycle progression of thyroid carcinoma cells from all histological derivations, but caused apoptotic cell death with DNA fragmentation, caspase activation, cell membrane disruption and LDH release.

### Sorafenib diminished MAP kinase and receptor tyrosine kinase activation in thyroid carcinoma cells

**Figure 4:**
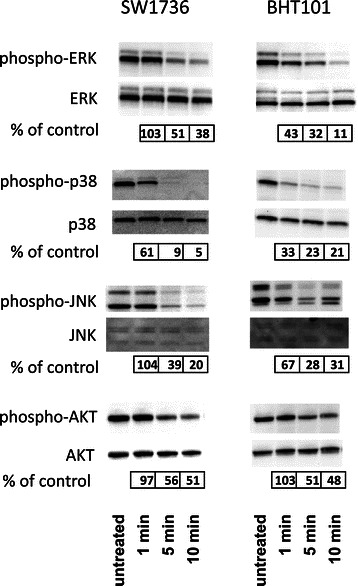
**Sorafenib suppressed phosphorylation of ERK, p38-MAP kinase, JNK and AKT in SW1736 and BHT101 thyroid carcinoma cells.** Cells were treated with 3 μM sorafenib for 1, 5 and 10 minutes. Whole-cell lysates were examined using western blot analysis. Expression of total protein was used as control. Signal intensities of phosphorylated proteins were corrected for signal intensities of total proteins and expressed as percent of untreated control.

To analyze which signaling pathways are targeted and disrupted in thyroid carcinoma cells by sorafenib, we assessed levels of phosphorylated members of the MAP kinase family and of receptor tyrosine kinases after sorafenib treatment for 10 minutes in BHT101, ML1, SW1736 and HTh7 cells. First we assessed phorphorylation of several common tyrosine kinase receptors using commercially available antibody arrays. Sorafenib inhibited the phosphorylation of VEGFR1 (FLT1), VEGFR2 (KDR) and PDGFRB in all four cell lines (Table 3). Sorafenib also inhibited phosphorylation of PDGFRA, which is expressed in the SW1736 and HTh7 anaplastic cells but not the BHT101 or ML1 cell lines (Table 3). Phosphorylation of VEGFR3 (FLT4) was significantly diminished in all cell lines but ML1. Sorafenib did not affect receptors of the EGFR family (EGFR, ERBB2, ERBB3 and ERBB4), the insulin receptor or the insulin-like growth factor receptor (IGF1R), which is in line with previous results of Wilhelm and co-workers (Wilhelm et al., 2006; Table 3). We investigated phosphorylation of AKT1, AKT2 and AKT3 as well as several MAP kinase family members, including JNK, p44/42 MAP kinase (ERK1/2) and p38 MAP kinase using dot blot analyses after sorafenib treatment of the same four cell lines for 10 minutes. Sorafenib significantly reduced phosphorylation of ERK1 in all four cell lines and ERK2 only in the two anaplastic cell lines, SW1736 and HTh7 (Table 4). Phosphorylation of the p38 alpha, −beta and -gamma isoforms was reduced in all four cell lines, while phosphorylation of the delta isoform of p38 MAP kinase was only diminished in SW1736 cells after sorafenib treatment (Table 4). Sorafenib significantly reduced JNK2 phosphorylation in all four cell lines, but reduced JNK1 and JNK3 phosphorylation only in HTh7 and ML1 cells, respectively. Phosphorylation of AKT1 and AKT2 was significantly reduced in all the four cell lines and AKT3 only in HTh7 and BHT101 cells by sorafenib (Table 4). Dot blot results were verified using western blotting of whole-cell lysates from SW1736 and BHT101 cells treated 1, 5 and 10 minutes with sorafenib. Western blots confirmed that sorafenib reduced the phosphorylation of ERK, p38 MAP kinase, JNK and AKT proteins within 5 to 10 minutes, while total protein remained constant (Figure 4). Taken together, sorafenib suppressed various intracellular signaling pathways in thyroid carcinoma cells treated in vitro, including VEGFRs, PDGFRs as well as various MAP kinase- and AKT-dependent pathways.

**Table 3:** **Dot blot analysis of tyrosine receptor kinase phosphorylation in SW1736, HTh7, BHT101 and ML1 cells after short-term (10 min) treatment with 3 μM sorafenib**

	% of untreated control			
Protein	SW1736	HTh7	BHT101	ML1
p-VEGFR1	41.3 ± 7.2*	27.2 ± 11.5*	62.5 ± 7.4*	70.3 ± 4.6*
p-VEGFR2	30.3 ± 10.2*	45.7 ± 8.8*	60.7 ± 5.7*	75.6 ± 6.7*
p-VEGFR3	44.2 ± 7.2*	67.9 ± 8.9*	68.3 ± 10.4*	94.1 ± 7.1
p-PDGFRA	46.6 ± 6.0*	28.5 ± 11.1*	n.e.	n.e.
p-PDGFRB	67.0 ± 8.1*	57.3 ± 6.8*	67.2 ± 10.9*	64.2 ± 9.0*
p-EGFR	102.8 ± 7.8	107.4 ± 10.4	105.0 ± 7.3	93.0 ± 11.4
p-ERBB2	96.3 ± 6.9	91.8 ± 9.9	101.5 ± 11.8	101.9 ± 9.3
p-ERBB3	111.3 ± 8.8	108.7 ± 7.4	95.3 ± 10.8	96.2 ± 11.6
p-ERBB4	107.5 ± 11.0	110.3 ± 12.4	94.9 ± 10.71	102.6 ± 7.8
p-insulinR	103.9 ± 6.9	97.7 ± 10.3	96.6 ± 8.8	112.1 ± 12.0
p-IGF1R	112.6 ± 10.5	101.8 ± 9.9	95.9 ± 8.9	110.3 ± 10.3

VEGFR: vascular endothelial growth factor receptor, PDGFR: platelet-derived growth factor receptor, EGFR: epidermal growth factor receptor, IGF1R: insulin-like growth factor 1 receptor, n.e.: not expressed.

Values for the respective protein compared to the vehicle-treated control ± standard deviation are depicted and represent 6-fold determinations. *indicates significant decrease (p<0.05, Student’s t-test).

**Table 4:** **Dot blot analysis of the activation of MAP kinase family members in SW1736, HTh7, BHT101 and ML1 cells after short-term treatment (10 min) with 3 μM sorafenib**

	% of untreated control			
Protein	SW1736	HTh7	BHT101	ML1
p-ERK1	54.2 ± 8.1*	67.1 ± 4.5*	45.4 ± 9.2*	82.0 ± 6.0*
p-ERK2	76.0 ± 5.4*	73.7 ± 3.7*	84.1 ± 5.7	88.1 ± 7.9
p-p38 alpha	38.9 ± 8.4*	44.4 ± 6.0*	32.2 ± 8.9*	61.3 ± 6.6*
p-p38 beta	68.6 ± 6.2*	80.7 ± 2.8*	75.9 ± 6.3*	76.7 ± 7.1*
p-p38 gamma	64.7 ± 5.9*	75.2 ± 5.4*	49.3 ± 5.7*	49.5 ± 8.3*
p-p38 delta	78.4 ± 5.0*	82.8 ± 8.8	83.6 ± 8.4	89.3 ± 6.9
p-JNK1	89.0 ± 7.7	79.1 ± 5.7*	86.7 ± 7.9	73.0 ± 9.8*
p-JNK2	61.2 ± 7.1*	75.1 ± 6.2*	77.5 ± 7.0*	63.2 ± 6.2*
p-JNK3	103.5 ± 7.7	86.3 ± 8.6	89.6 ± 5.4	69.2 ± 6.8*
p-AKT1	72.1 ± 5.9*	80.2 ± 5.8*	83.4 ± 6.3*	75.7 ± 5.5*
p-AKT2	74.7 ± 9.2*	80.0 ± 7.2*	81.9 ± 7.0*	77.4 ± 6.1*
p-AKT3	92.3 ± 8.3	77.6 ± 6.2*	81.5 ± 5.6*	86.6 ± 9.7

*Abbreviations*: *ERK* extracellular-signal regulated kinase, *p38* p38 mitogen-activated kinase, *JNK* c-jun N-terminal kinase, *AKT* AKT/protein kinase B.

Values for the respective protein compared to the vehicle-treated control ± standard deviation are depicted and represent 6-fold determinations. *indicates significant decrease (p<0.05, Student’s t-test).

## Discussion

Here we present a detailed analysis of kinase inhibition, effects on the cell cycle and apoptosis induction by the BRAF- and multikinase inhibitor, sorafenib, in thyroid carcinoma cell lines of various histological subtypes with and without activating *BRAF*^*V600E*^ mutations. The effects of sorafenib on various intracellular signaling molecules were studied to evaluate more specific treatment options in patients with dedifferentiated thyroid carcinomas patients. We assessed the *BRAF*^*V600E*^ mutational status for all 12 cell lines used in this study. The activating *BRAF*^*V600E*^ mutation was only detected in two of the three papillary cell lines (BHT101 and B-CPAP) and in one of the four cell lines (SW1736) as previously detected and reported [12,48]. The HTh7, C643 and HTh83 anaplastic cell lines, the TPC papillary cell line and the FTC133, FTC236, FTC238, ML1 and TT2609 follicular cell lines harbored only wildtype alleles for *BRAF*. These findings fit well with experimental and pathological evidence indicating an involvement of *BRAF* mutation in the pathogenesis of about 50% of PTCs and the progression of PTC to ATC, but no occurrence of *BRAF* mutations in FTC [9,10,12,49].

Proliferation of all cell lines was inhibited by sorafenib within the 48 h treatment period. To our knowledge, ours is the first report about the inhibitory effects of sorafenib not only on cell lines derived from PTCs and ATCs, but also from FTCs. It is in good agreement with recent clinical findings in the phase III DECISION trial of sorafenib in patients with iodine-refractory thyroid cancer, where positive effects of sorafenib on progression-free survival was found in all clinical and genetic biomarker subgroups [35]. In contrast, Kloos et al. [29] reported better clinical responses to sorafenib in patients with PTC than in those with FTC (in patients with PTC partial response in 15% and stable disease in approx. 65% of patients, in patients with FTC no partial response and stable disease in 80% of patients, in patients with ATC stable disease in 25% of patients) [29]. IC50 values for sorafenib in the various cell lines investigated in the present study ranged from 1.85 μM to 4.2 μM, which correspond to the lower range of achievable plasma levels. A daily dose of 400 mg sorafenib administered orally or 2 doses of 200 mg per day resulted in mean plasma levels of 20 μM in patients during a phase I trial [50]. The two papillary cell lines BCPAP and BHT101 with the *BRAF*^*V600E*^ mutations had the lowest IC50 values for sorafenib, while a slightly higher IC50 value was calculated for the TPC1 papillary cell line, which harbors no *BRAF*^*V600E*^ mutation, but the RET/PTC1 rearrangement [51]. Cell lines derived from FTCs and ATCs responded similarly in this study, as evidenced by IC50 values within a relatively narrow range. These IC50 values for FTC and ATC cell lines were slightly higher than those for PTC cell lines, but still comparable to the lower range of plasma concentrations that are achieved in patients [50]. Recently, Cohen et al. reported on a synergistic effect of sorafenib treatment with withaferin A in the B-CPAP and SW1736 thyroid carcinoma cell lines [52]. IC50 values for sorafenib treatment alone were 6.3 μM (B-CPAP) and 7.6 μM (SW1736). Although the IC50 values we report are somewhat lower, with 1.85 μM for B-CPAP and 3.25 μM for SW1736, they are in the same order of magnitude with BCPAP being the more sensitive cell line. The IC50 values we report are close to the IC50 values in the 1 μM-range reported by Salvatore et al. for sorafenib treatment of the FRO, ARO, KAT4 and NPA ATC cell lines harboring *BRAF*^*V600E*^ mutations [25]. IC50 values for thyroid carcinoma cell lines are also very close to those reported in the literature for hepatocellular carcinoma cell lines (4.5 and 6.3 μM) [24] and melanoma cell lines (~5 μM) [53,54] treated with sorafenib.

The lowest sorafenib concentration that led to a significant antiproliferative effect in our study was the lowest in three cell lines without *BRAF*^*V600E*^ mutations: In the fast growing C643 and FTC238 cells, significant effects on cell number were achieved with 0.01 μM. In papillary TPC1 cells significant effects were achieved with 0.05 μM sorafenib while in BHT101 and B-CPAP cells significant inhibition was achieved with 1.0 μM as the lowest concentration. The molecular reasons for these effects are unclear, and may stem from the multikinase-inhibitor activity of sorafenib. It also points to positive effects that may be achieved by sorafenib even in low concentrations due to side effects during sorafenib treatment.

Sorafenib induced cell death in all 12 thyroid carcinoma cell lines investigated here, regardless of histological derivation or the presence of the activating *BRAF*^*V600E*^ mutation. We detected increases in the percentage of cells in subG1 for all cell lines, and different influences on cell cycle progression depending on the cell line. Sorafenib induced a larger proportion of cells of papillary and anaplastic cell lines to enter subG1 than of follicular cell lines, indicating that sorafenib has a different kind of intracellular effect on DNA fragmentation in follicular cell lines. Analysis of the proportion of the treated culture that did not enter subG1 revealed that G1 arrest was induced in all PTC and two of four ATC cell lines, while S phase arrest with G1 decrease was induced in one ATC and three FTC cell lines. The TT2609 follicular cell line showed no alteration in cell cycle phases of the living proportion of the culture. These data concerning the G1 arrest together with the occurrence of a subG1 peak are in agreement with literature data on the TPC1 papillary thyroid carcinoma cell line, the TT medullary thyroid carcinoma cell line [26] and the ARO anaplastic thyroid carcinoma cell line treated with sorafenib concentrations in a similar concentration range as we used in vitro [25]. On the other hand, Liu et al. observed a decrease in the number of cells in G1 and an increase of cells in S phase in HepG2 hepatocellular carcinoma cells treated with sorafenib [24]. These results indicate that sorafenib affects the cell cycle differently depending on the cellular background. Since all papillary cell lines we examined as well as the SW1736 anaplastic cell line harboring the activating *BRAF*^*V600E*^ mutation and HTh7 cells arrested in G1 after sorafenib treatment, one may speculate that inhibition of the overactivated RAF-MAP kinase pathway in these cells contributes to the G1 arrest while other, yet unidentified, molecular effects lead to arrest in the S or G2/M phases in the other cell lines.

We further characterized the mechanism of cell death in detail in four thyroid carcinoma cell lines. We chose BHT101 as an example of a PTC cell line with a heterozygous *BRAF*^*V600E*^ mutation, ML1 as a FTC cell line, SW1736 as an ATC cell line harboring the *BRAF*^*V600E*^ mutation and HTh7 as an ATC cell line with wildtype *BRAF*. All four cell lines showed marked LDH release into the medium after 14 and 24 hours of treatment, confirming plasma membrane breakdown and release of cytoplasmic contents. Apoptotic cell death was confirmed by the increased activity of caspases 3 and 7 in all four cells lines. Interestingly, values for LDH release and caspase activities were in the same magnitude in all four cell lines. LDH release was slightly, but not significantly higher in SW1736 and ML1 cells compared to BHT101 and HTh7 cells. In contrast, caspase 3 and 7 activities were slightly, but not significantly elevated in *BRAF*^*V600E*^ mutation-positive SW1736 and BHT101 cells compared to HTh7 and ML1 cells. These results are in contrast to recently reported results by Preto and coworkers [55], who reported that sorafenib treatment only significantly induced apoptosis in anaplastic thyroid cells harboring a homozygous *BRAF*^*V600E*^ mutation (8505C cell line), but not in thyroid carcinoma cells with wildtype *BRAF* (C643 and TPC1 cell lines). Preto et al. used the TUNEL assay to quantify apoptosis, and since TUNEL detects DNA fragments directly, it corresponds methodically to quantification of the subG1 peaks in our study. Kim et al. [27] on the other hand observed no correlation between the inhibition of cell proliferation or apoptotic induction (measured as subG1 peak) and the presence of the activating *BRAF*^*V600E*^ mutation in five anaplastic thyroid carcinoma cell lines treated with sorafenib [27] which is in accordance with our results. Analysis of caspase activity, however, reflects other mechanisms of cellular death than investigation of DNA fragmentation by subG1 peak analysis and the TUNEL assay. Caspases are key effector proteins in apoptosis that initiate systemic structural disassembly in dying cells and have a multitude of intracellular substrates (Review [56]). Concerning the effects of the *BRAF*^*V600E*^ mutation to apoptosis resistance, Lee et al. recently showed in the nontransformed PCCl3 rat thyroid cells and in the cervical carcinoma cell line, HeLa, that transfection with an inducible *BRAF*^*V600E*^ construct mediates resistance to mitochondrial-induced apoptosis following sorafenib treatment [57]. Overall, the effect of *BRAF*^*V600E*^ mutation on apoptosis induction appears to be different in various cellular contexts. In our experimental setting, apoptosis induction and membrane disruption after sorafenib treatment was not significantly influenced by the histological origin of and BRAF mutational status of thyroid carcinoma cells.

We also examined the of sorafenib on phosphorylation of specific tyrosine kinase receptors in selected thyroid carcinoma cell lines to better assess the impact of differing cellular backgrounds from histological derivation and the presence of the activating *BRAF*^*V600E*^ mutation. Screening of receptor tyrosine kinase receptor activation to identify the inhibitory mechanism of sorafenib exhibited similar results in all four cell lines. Sorafenib inhibited phosphorylation of VEGFRs and PDGFRs receptors, but did not affect phosphorylation of insulin receptors, IGF1R and the EGF family of receptors in thyroid carcinoma cells. These results fit well with results reported for other cell types [22,58]. Sorafenib treatment in vivo has been shown to also inhibit these tyrosine kinase receptors in endothelial cells and, thus, be capable of inhibiting tumor vascularization [20]. In vitro biochemical assay showed that sorafenib directly inhibits the RAF1, BRAF and oncogenic BRAF^V600E^ kinases, but has no significant inhibitory effects on MEK, ERK, AKT and other signaling pathways [22]. Complex crosstalk mechanisms can occur between signaling pathways in the cell, however, that lead to stepwise activation of different pathways depending on the cellular context. The ERK kinase inhibition we observed and that has been described by others in thyroid carcinoma cells [24-26] can easily be explained by inhibition of receptor tyrosine kinases or the RAF1 or BRAF molecule in the RAF-MEK-ERK kinase cascade [19]. We observed a more pronounced inhibition of ERK1 than ERK2 by sorafenib in thyroid carcinoma cells. We can only speculate about the molecular mechanism behind this effect at this time. Differential regulation of ERK1 and ERK2 was already described in other systems (Review [59]). The diminished AKT phosphorylation we observed in thyroid carcinoma cells, beside possible direct effects, may also be the result of receptor tyrosine kinase inhibition by sorafenib and has recently been described in prostate cancer cells [60]. Inhibition of p38 MAP kinase by sorafenib has already been reported in cell-free kinase assays [20]. An inhibition of p38 MAP kinase and JNK by sorafenib comparable to that in our cells was also reported in human hepatoma cell lines [61]. JNK phosphorylation was also reported to be suppressed in endothelial cells after sorafenib treatment [62].

## Conclusions

In summary, our results demonstrate that the BRAF- and multikinase inhibitor, sorafenib, exhibited various inhibitory effects on intracellular signaling pathways in thyroid carcinoma cells and caused cell death and cell cycle arrest. Since sorafenib was effective against all thyroid carcinoma cell lines, independent of their histological derivation or the presence of an activating *BRAF* mutation, these in vitro data are in accordance with data collected so far from patients with various thyroid carcinoma subtypes treated with sorafenib in clinical trials. Our data support the hypothesis that sorafenib may be effective against dedifferentiated thyroid cancers of all histological subtypes and regardless of their *BRAF* status. The intracellular targets of sorafenib in thyroid carcinoma cells described in this study may allow the development of more specific therapeutic intervention with less side effects.

## Abbreviations

ATC: Anaplastic thyroid carcinoma
BSA: Bovine serum albumin
EGFR: Epidermal growth factor receptor
ERK: Extracellular-signal regulated kinase
FBS: Fetal bovine serum
FTC: Follicular thyroid carcinoma
IGFR: Insulin like growth factor receptor
MAP kinase: Mitogen activated protein kinase
JNK kinase: c-Jun N-terminal kinase
LDH: Lactate dehydrogenase
MEK: Mitogen-activated protein kinase kinase
PDGFR: Platelet derived growth factor receptor
PDTC: Poorly differentiated thyroid carcinoma
PTC: Papillary thyroid carcinoma
VEGFR: Vascular endothelial growth factor receptor
